# Unveiling Bacterial Interactions through Multidimensional Scaling and Dynamics Modeling

**DOI:** 10.1038/srep18396

**Published:** 2015-12-16

**Authors:** Pedro Dorado-Morales, Cristina Vilanova, Carlos P. Garay, Jose Manuel Martí, Manuel Porcar

**Affiliations:** 1 Cavanilles Institute of Biodiversity and Evolutionary Biology (Universitat de València), 46020 Valencia, Spain; 2 Fundació General de la Universitat de València, Spain; 3 Instituto de Física Corpuscular, CSIC-UVEG, 46071, Valencia, Spain

## Abstract

We propose a new strategy to identify and visualize bacterial consortia by conducting replicated culturing of environmental samples coupled with high-throughput sequencing and multidimensional scaling analysis, followed by identification of bacteria-bacteria correlations and interactions. We conducted a proof of concept assay with pine-tree resin-based media in ten replicates, which allowed detecting and visualizing dynamical bacterial associations in the form of statistically significant and yet biologically relevant bacterial consortia.

There is a growing interest on disentangling the complexity of microbial interactions in order to both optimize reactions performed by natural consortia and to pave the way towards the development of synthetic consortia with improved biotechnological properties^1^,^2^. Despite the enormous amount of metagenomic data on both natural and artificial microbial ecosystems, bacterial consortia are not necessarily deduced from those data. In fact, the flexibility of the bacterial interactions, the lack of replicated assays and/or biases associated with different DNA isolation technologies and taxonomic bioinformatics tools hamper the clear identification of bacterial consortia. We propose here a holistic approach aiming at identifying bacterial interactions in laboratory-selected microbial complex cultures. The method requires multi-replicated taxonomic data on independent subcultures, and high-throughput sequencing-based taxonomic data. From this data matrix, randomness of replicates can be verified, linear correlations can be visualized and interactions can emerge from statistical correlations. The whole procedure can be summarized as follows:

1. Taxonomic data from multi-replicated, independent assays is obtained.
2. Fluctuation scaling of replicates, i.e. slope of Taylor’s law, is validated for the samples of the first time step against the expectation of a Poisson-distributed selection.
3. Linear correlation coefficients are determined, converted into distances and displayed by multidimensional scaling.
4. Interaction matrix is inferred from the correlation matrix using the discrete Lotka-Volterra model with relative abundances^3^.

As a proof of concept, we chose to analyze independent laboratory cultures grown on a natural, recalcitrant compound. A range of recalcitrant substrates, from synthetic dyes to polycyclic aromatic hydrocarbons, polychlorinated biphenyls, and other organic pollutants can be efficiently degraded by mixed microbial cultures combining catabolic enzyme activities of individual consortium members^4^. Therefore, a carbon source that requires complex pathways for degradation is expected to shape the structure of the microbial community and behave as a strong selection pressure towards the establishment of microbial consortia with biodegradation properties.

In a previous work, we characterized the cultivable microbial communities present in coniferous resin, and detected a rather diverse microbial community, including several fungal and bacterial strains with potential use in bioremediation as deduced from their ability for the degradation of different terpenic compounds^5^. In the proof of concept we present here, we used pine-tree resin as the main carbon source of a resin-rich semi-synthetic medium (prepared as described in our previous report^5^), which we inoculated with environmental resin in ten independent subcultures. We designed this multi-replicate experimental evolution assay to address three main issues: i) Time-course variation in biodiversity fate (does sub-culturing in a recalcitrant compound lead to taxonomic impoverishment?); ii) reproducibility of the selection process (do the replicates behave as such?); and iii) strain-to-strain bacterial interactions: can microbial interactions be deduced from a close analysis of the biodiversity dataset?

## Results and Discussion

### 16S profiles-based monitoring of bacterial populations throughout the experiment

**Figure 1:**
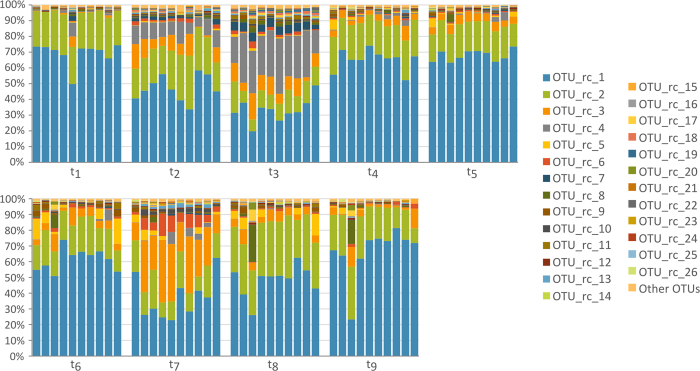
Bacterial biodiversity as deduced from 16S amplicon high-throughput sequencing of ten replicated culture lines subcultured in parallel nine times (t_1_ to t_9_). (**A**) pool of pine-tree resin samples was used to inoculate ten independent minimum broth media supplemented with resin as the main carbon source. Sub-culturing was carried out after 4 (t_1_-t_2_, t_2_-t_3_), 3 (t_3_-t_4_, t_7_-t_8_), or 2 days (t_4_-t_5_, t_5_-t_6_, t_6_-t_7_, t_8_-t_9_) and the complete assay lasted 24 days. Each bar represents an independent experiment at a given time. The taxonomic assignation of each OTU is available in Table 1.

Ten replicated cultures of resin samples in resin-rich semisynthetic medium were set up and independently subcultured (nine times) during 24 days, as described in the Methods section. Rather than within replicates, the taxonomic profiles displayed statistically significant variations within time steps (p-value = 0.01, see Methods). Indeed, significant alterations (p-value = 0.02) were found at times 2, 3 and 7, in correlation with changes in the number of days between subculturing events (Fig. 1). For nearly all time points and replicates, *Pseudomonas* sp. (OTU_rc_1) followed by an enterobacterium (OTU_rc_2) were the most frequent species, being overwhelmingly present at subculturing steps 1, 4, 5, 6, 8, and 9. A species belonging to the Xanthomonadaceae family (OTU_rc_3) and *Comamonas terrigena* (OTU_rc_4) were also found at relatively high rates, particularly at subculturing steps 3 (*C. terrigena*), 7 (Xanthomonadaceae species) and 2 (both). Table 1 shows the taxonomical assignations for the 26 most abundant OTUs. Twenty-two other relatively frequent OTUs were detected at a comparatively lower, but still significant frequency (more than 100 counts), and as many as 87 different OTUs could be detected in total.

In order to find out whether sub-culturing was associated with a change in sample biodiversity, Shannon and Simpson indexes, as well as Species Richness were calculated for each sample. Richness index was virtually constant throughout the whole experiment; whereas Shannon and Simpson indexes fluctuated in time: they increased between subculturing steps 1 and 3, then dropped, and then increased again until subculturing step 7, showing a slightly decreasing trend at the end of the experiment (Supplementary Fig. 1). These fluctuations coincided with changes in the number of days between subculturing steps, suggesting, again, the influence of this factor in community composition.

### Identification of microbial consortia

**Figure 2:**
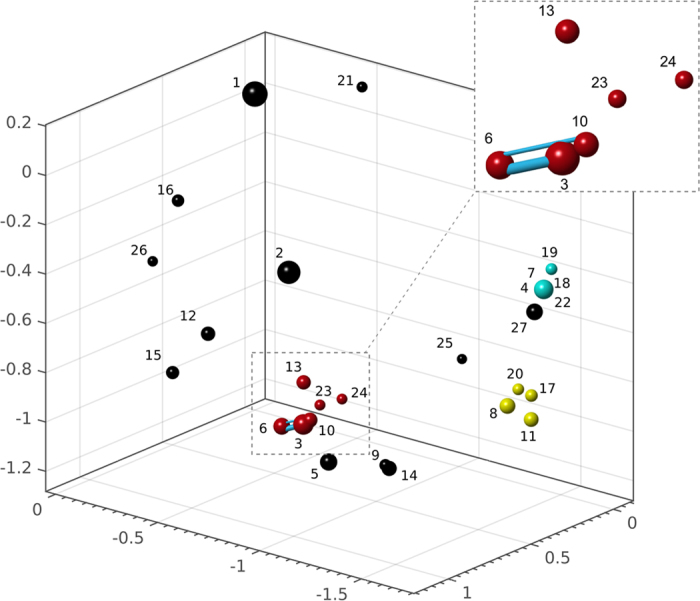
Correlations and interactions among OTUs. Representation of the 3D positions of identified OTUs in our samples, where the distance corresponds to the amount of linear statistical correlation. Smaller distances mean stronger positive correlation and larger distances show stronger negative correlation. Sizes of the spheres are proportional to the relative abundances in logarithmic scale, while colors indicate putative bacterial consortia (OTU groups positively correlated). Blue bonds show the strength of significant positive interactions linking two OTUs, where the width of the bonds linearly scales with the relative strength of the interaction. Bonds linking OTUs 4–7 and 3–10 are hidden in this 3D perspective. The plotting code is available as Supplementary Data. The main consortia are colored as follows: yellow (*Pseudomonas alcaligenes*, *Acinetobacter* sp., Moraxellaceae OTU, Comamonadaceae OTU); blue (two species of the genus *Comamonas*, *Zooglea* sp., Neisseriaceae OTU, Burkholderiales OTU); and red (three species of the *Stenotrophomonas* genus, *Pseudoxanthomonas mexicana, Luteimonas* sp.). OTUs are numbered according to Table 1.

Components of the consortia were identified through a three-phase analysis of a selected pool of data consisting of correlated time-series of relative abundances for the 26 more frequent OTUs in our samples. We first characterized the randomness of our ten subcultures at the first sampled time. The fluctuation scaling of the Taylor law (log of the standard deviation versus the mean frequency of each OTU) of our samples was 0.60 ± 0.04 (Supplementary Fig. 2), which is consistent with Poisson-distributed replica (0.5). Data of each replicated time series were analyzed both independently and together, as a mixture of all ten replica. The linear correlation coefficients among OTUs were calculated and the correlation matrix was converted into a distance matrix by shifting the correlation coefficients by two units^6^,^7^. Multidimensional scaling (MDS) was used to display the positions of the OTUs in a given dimension, where distances are well preserved. The stress value of our MDS solution was 0.092, which falls between “fair” and “good” goodness of fit according to Kruskal’s criterion^8^. We show the distances among OTUs by statistical correlation in Fig. 2, where the size of the spheres scale logarithmically with the relative abundance. We determined the statistical significance of the inferred correlations by performing permutation tests and also by simulating 10,000 experiments with the assumption that the counts have lognormal distribution (both approaches yielded similar results, data not shown). Three main consortia (OTU groups positively correlated) were observed: OTUs 3, 6, 10, 13, 23, and 24; OTUs 4, 7, 18, 19, and 22; and OTUs 8, 11, 17, and 20. The first association involved only gamma-proteobacteria (mainly, three species of the *Stenotrophomonas* genus, and also *Pseudoxanthomonas mexicana,* and *Luteimonas* sp.); the second comprised only beta-proteobacteria (two species of the genus *Comamonas*, *Zooglea* sp., and two species belonging to the Neisseriaceae family and the Burkholderiales order, respectively); and, finally, the third one was composed of three gamma-proteobacteria (*Pseudomonas alcaligenes*, *Acinetobacter* sp., and a species of the Moraxellaceae family) and one beta-proteobacterium of the Comamonadaceae family. A significant negative correlation was found between OTU 12 (*Pseudomonas stutzeri*) and the consortia composed of OTUs 4, 7, 18, 19, and 22.

Statistical correlation does not imply interaction^3^. We inferred the interaction matrix by using the discrete Lotka-Volterra model with relative abundances, a forward stepwise regression to include the strongest interactions and a bootstrap aggregation method to cure instabilities. Four statistically significant, positive interactions (Fig. 2) were found between OTUs 4 and 7 (*Comamonas terrigena* and *Comamonas* sp.); OTUs 3 and 10 (*Pseudoxanthomonas mexicana* and other Xanthomonadaceae species); OTUs 3 and 6 (the same Xanthomonadaceae species and *Stenotrophomonas geniculata*); and OTUs 6 and 10 (*S. geniculata* and *P. mexicana*).

Interestingly, the three bacterial associations detected in this work include well known terpenoid degraders, particularly *Pseudomonas*^9^ and, to a lesser extent, *Comamonas*^10^ and *Stenotrophomonas*^11^. The resolution of the taxonomic assignations for the OTUs detected in this work poses difficulties in identifying putative metabolic complementation established among the members of each consortia. However, the associations unveiled with our analysis have been experimentally reported in previous studies. Particularly, *Pseudomonas*, *Comamonas*, and *Acinetobacter* species (the main taxa found in the third association) are known to form natural consortia able to degrade a range of recalcitrant compounds such as hydrocarbons^12^, phenolic compounds^13^,^14^ and herbicides^15^. Interestingly, the degradation of these compounds involves similar steps (such as the cleavage of aromatic rings by dioxygenase enzymes^16^) to those of the degradation pathway of the terpenes present in pinetree resin. *Pseudoxanthomonas* and *Stenotrophomas* species (detected in the first association and displaying strong, positive interaction) have also been reported as part of other consortia involved in the bioremediation of TNT-contaminated soils^17^. Further genomic-scale analyses are needed to better understand the relationships established at the molecular level among the members of the microbial consortia unveiled in this study.

This work shows how microbial consortia can be visualized after a robust analysis of their relative frequencies in multireplicated cultures. The resulting plot can be very helpful to rapidly identify bacterial key-players and consortia in a wide range of bioprocesses. From the proof of concept of our method, we can conclude that unambiguous consortia can be detected within a pool of coexisting species on the basis not only of their relative abundance, but also by statistical correlation and verification of inferred biological interactions.

## Methods

### Culture medium and growth conditions

A pine-tree resin-based medium previously described^5^ containing 0.1% (w/v) of resin as the main carbon source was used for microbial culturing. A starter culture of environmental resin in the resin-rich semi-synthetic medium was set up, and ten 5 mL-aliquots were taken from it and incubated independently for 4 days (time series 1, t_1_). Then, a 50 μL aliquot of each culture was inoculated in a new set of ten independent tubes containing fresh medium, and incubated for another 4 days (t_2_). New 50 μL aliquots were taken from the second round of cultivation, and the whole process was repeated a total of 9 times. Since previous observations in the laboratory proved that few sub-culturing steps resulted in an accelerated microbial growth (resin colloids were consumed faster), the time between sub-cultures was shortened throughout the experiment from 4 to 2 days in order to promote the selection of efficient resin-degrading consortia. Tubes were grown for 4 days in t_1_-t_2_ and t_2_-t_3_; 3 days in t_3_-t_4;_ and 2 days in t_4_-t_5_, t_5_-t_6_, t_6_-t_7_, and t_8_-t_9._ Three days of incubation in t_7_-t_8_ were applied to compensate an unexpected decrease (from 30 to 28 °C) in the temperature of the incubator. The complete assay lasted 24 days, and aliquots were taken at every sub-culturing step to obtain a total of 90 (10 replicated cultures x 9 sub-culturing steps) culture samples.

### DNA isolation and quantification

A two mL aliquot of each culture was sampled and resin colloids from the culture medium were pelleted by mild centrifugation (800 g, 5 min). The supernatant was transferred to a new tube, cells were harvested at 11,000 g for 3 min and washed twice with sterile PBS buffer (NaCl 8 g/L, KCl 0.2 g/L, Na_2_HPO_4_ 1.44 g/L, KH_2_PO_4_ 0.24 g/L, pH adjusted to 7.4). Then, DNA was isolated with a standard protocol consisting of alkaline lysis followed by precipitation with potassium acetate and isopropanol. The quality of the DNA was finally checked on a 0.8% (w/v) agarose gel and quantified with Nanodrop-1000 Spectophotometer (Thermo Scientific, Wilmington, DE).

### PCR amplification

A 500 bp fragment of the V1–V3 hypervariable region of the 16S ribosomal RNA gene was PCR-amplified from all the samples with primers 28F (5′-GAG TTT GAT CNT GGC TCA G-3′) and 519R (5′-GTN TTA CNG CGG CKG CTG-3′). A short (9–11 nucleotides) barcode sequence followed by a four-nucleotide spacer (CGAT) was included at the 5′ end of the oligonucleotides used as forward primers to enable assignment of sequences to samples after high-throughput sequencing. All the amplifications were performed under the following thermal cycling conditions: initial denaturing at 95 °C for 5 min, followed by 35 cycles of denaturing at 95 °C for 30 s, annealing at 54 °C for 30 s, and extension at 72 °C for 1 min, finalized by a 10-min elongation at 72 °C. The resulting amplicons were checked on a 0.8% (w/v) agarose gel and purified by precipitation with 3M potassium acetate (pH = 5) and isopropanol. Pure amplicons were quantified with the Qubit® 2.0 Fluorometer (Invitrogen, Carlsbad, CA, USA) and an equimolar pool of amplicons was prepared from all the samples.

### Sequencing

A sequencing library was constructed with 100 ng of the pool by amplicon fusion (Ion Plus Fragment Library Kit, MAN0006846, Life Technologies). The library was quantified with the Agilent 2100 Bioanalizer (Agilent Technologies Inc, Palo Alto, California) prior to clonal amplification. Emulsion PCRs were carried out applying the Ion PGM Template OT2 400 kit as described in the user-guide (MAN0007218, Revision 3.0 Lifetechnologies) provided by the manufacturer. Finally, the library was sequenced in an Ion 318 Chip v2 on a Personal Genome Machine (PGM IonTorrentTM, Lifetechnologies) at Lifesequencing S.L (Lifesequencing, Valencia, Spain), using the Ion PGM Sequencing 400 kit following the manufacturer’s protocol (Publication Number MAN0007242, Revision 2.0, Lifetechnologies).

### Bioinformatic analysis

Raw sequences were filtered to remove short (<200 bp) and low quality (<Q10) reads with the NextGENe® Software for Ion Torrent PGM™ System, and the resulting sequences were processed with the QIIME package^18^ according to the following pipeline. First, a mapping file was generated by assigning reads to samples according to barcode sequences (allowing a maximum of 1 mismatch in primer search and 1 mismatch in barcode search). Second, an OTU table was generated with the *uclust* algorithm, using a similarity threshold of 0.97, corresponding to species-level OTUs. Then, representative sequences were picked from each OTU and classified with the BLAST algorithm (e-value <1e-05) against the 16S Greengenes database (version 13_8). Finally, an OTU table containing the taxonomical identifications and the absolute abundance of each OTU in every sample was built.

In order to determine whether microbial composition significantly changed among replica and/or with time (variation between sub-culturing steps and/or variation of the time between these steps), a matrix of Bray-Curtis beta-diversity dissimilarities was calculated with QIIME, and an ANOSIM test with 999 permutations was carried out for each hypothesis.

### Biostatistical analysis

#### Multidimensional Scaling

The OTU table containing the frequency of each OTU in every sample was used to compute the linear correlation matrix of the fluctuations observed in the 9 time series with the SparCC software^6^. Correlations were linearly converted into positive distances, so that positive full correlation was d = 0 and negative full correlation was d = 2. A matrix of distances, if all are precisely know, can be uniquely converted into positions up to dimension, translation and rotational symmetry. In our study, we chose non-metric multidimensional scaling in the MATLAB® function *mdscale*. We devised using three dimensions (3D) in the plot as a requirement to minimize 2D image ambiguity effects in the visualization of correlated, uncorrelated and anticorrelated taxa.

#### Interaction

For a determined taxonomical level, the discrete time Lotka-Volterra model (dLV) relates the abundance of taxon *i* at an arbitrary future time *t + δt* to the abundances of all the taxa at the present time *t*. The interactions^19^ are described by interaction coefficients *c*_*ij*_ that describe the influence of taxon *j* on the abundance of taxon *i*. If the number of taxons is large, the dynamics of relative abundances can be described by a modified dLV model generalized to include stochasticity, like environmental and demographic stochastic effects^3^ (Equation 1):


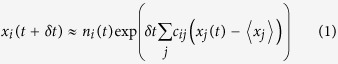


where *η*_*i*_*(t)* is a log-normally distributed multiplicative noise, *x*_*i*_ is the relative abundance of taxon *i* and *x*_*j*_ is the equilibrium abundance of taxon *j* and is set by the carrying capacity of the environment. In this problem, the interaction coefficients are known up to an arbitrary multiplicative constant and the design matrix is singular. We inferred the interaction coefficients with the LIMITS algorithm^3^.

## Additional Information

**How to cite this article**: Dorado-Morales, P. *et al.* Unveiling Bacterial Interactions through Multidimensional Scaling and Dynamics Modeling. *Sci. Rep.* **5**, 18396; doi: 10.1038/srep18396 (2015).

## Supplementary Material

Supplementary Information

## Figures and Tables

**Table 1:** Taxonomical classification obtained for the OTUs analyzed in this study.

	Taxonomical classification				
ID	Class	Order	Family	Genus	Species
OTU_rc_1	γ-proteobacteria	Pseudomonadales	Pseudomonadaceae	*Pseudomonas*	*—*
OTU_rc_2	γ-proteobacteria	Enterobacteriales	Enterobacteriaceae	*—*	*—*
OTU_rc_3	γ-proteobacteria	Xanthomonadales	Xanthomonadaceae	*—*	*—*
OTU_rc_4	β-proteobacteria	Burkholderiales	Comamonadaceae	*Comamonas*	*terrigena*
OTU_rc_5	β-proteobacteria	Burkholderiales	Alcaligenaceae	*Achromobacter*	*—*
OTU_rc_6	γ-proteobacteria	Xanthomonadales	Xanthomonadaceae	*Stenotrophomonas*	*geniculata*
OTU_rc_7	β-proteobacteria	Burkholderiales	Comamonadaceae	*Comamonas*	*—*
OTU_rc_8	γ-proteobacteria	Pseudomonadales	Moraxellaceae	*—*	*—*
OTU_rc_9	β-proteobacteria	Burkholderiales	Comamonadaceae	*Delftia*	*—*
OTU_rc_10	γ-proteobacteria	Xanthomonadales	Xanthomonadaceae	*Pseudoxanthomonas*	*mexicana*
OTU_rc_11	β-proteobacteria	Burkholderiales	Comamonadaceae	*—*	*—*
OTU_rc_12	γ-proteobacteria	Pseudomonadales	Pseudomonadaceae	*Pseudomonas*	*stutzeri*
OTU_rc_13	γ-proteobacteria	Xanthomonadales	Xanthomonadaceae	*Stenotrophomonas*	*—*
OTU_rc_14	β-proteobacteria	Burkholderiales	Alcaligenaceae	*—*	*—*
OTU_rc_15	γ-proteobacteria	Pseudomonadales	Pseudomonadaceae	*—*	*—*
OTU_rc_16	γ-proteobacteria	Pseudomonadales	Pseudomonadaceae	*Pseudomonas*	*viridiflava*
OTU_rc_17	γ-proteobacteria	Pseudomonadales	Pseudomonadaceae	*Pseudomonas*	*alcaligenes*
OTU_rc_18	β-proteobacteria	Neisseriales	Neisseriaceae	*—*	*—*
OTU_rc_19	β-proteobacteria	Rhodocyclales	Rhodocyclaceae	*Zoogloea*	*—*
OTU_rc_20	γ-proteobacteria	Pseudomonadales	Moraxellaceae	*Acinetobacter*	*—*
OTU_rc_21	γ-proteobacteria	Aeromonadales	Aeromonadaceae	*—*	*—*
OTU_rc_22	β-proteobacteria	Burkholderiales	*—*	*—*	*—*
OTU_rc_23	γ-proteobacteria	Xanthomonadales	Xanthomonadaceae	*Luteimonas*	*—*
OTU_rc_24	γ-proteobacteria	Xanthomonadales	Xanthomonadaceae	*Stenotrophomonas*	*acidaminiphila*
OTU_rc_25	γ-proteobacteria	Alteromonadales	Shewanellaceae	*Shewanella*	*—*
OTU_rc_26	Sphingobacteria	Sphingobacteriales	Sphingobacteriaceae	*Sphingobacterium*	*multivorum*
