# Supplementary material for: Unveiling Bacterial Interactions through Multidimensional Scaling and Dynamics Modeling
Source: Sci Rep. 2015 Dec 16;5:18396. doi: 10.1038/srep18396 (PMC4680887; doi:10.1038/srep18396)
Supplement: Supplementary Information [file srep18396-s1.pdf]

Unveiling Bacterial Interactions through Multidimensional Scaling and Dynamics Modeling

Pedro Dorado-Morales[1], Cristina Vilanova[1], Carlos P. Garay[3], Jose Manuel Martí[3], and Manuel Porcar[1,2*]

[1]Cavanilles Institute of Biodiversity and Evolutionary Biology (Universitat de València), 46020 Valencia, Spain.

[2]Fundació General de la Universitat de València, Spain.

[3]Instituto de Física Corpuscular, CSIC-UVEG, 46071, Valencia, Spain.

Supplementary information

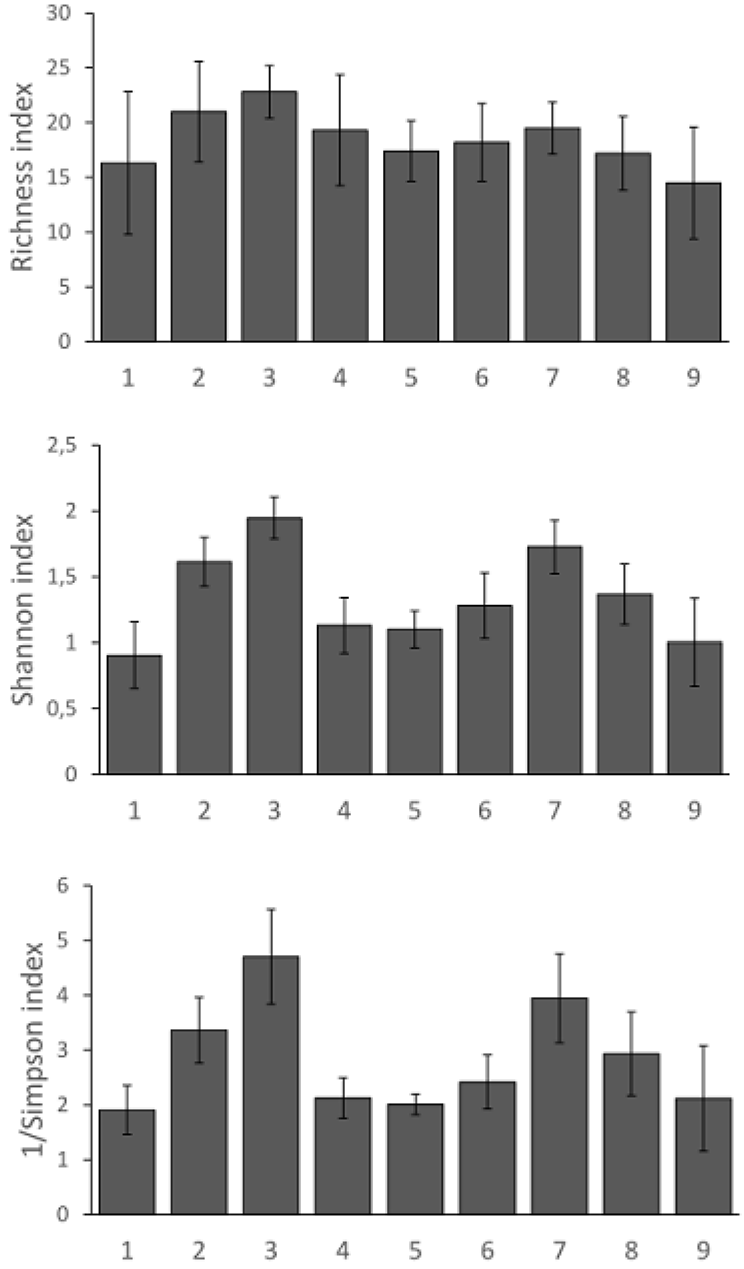

Supplementary Figure 1. Evolution of biodiversity indexes throughout the experiment. Numbers in the horizontal axis correspond to each subculturing step.

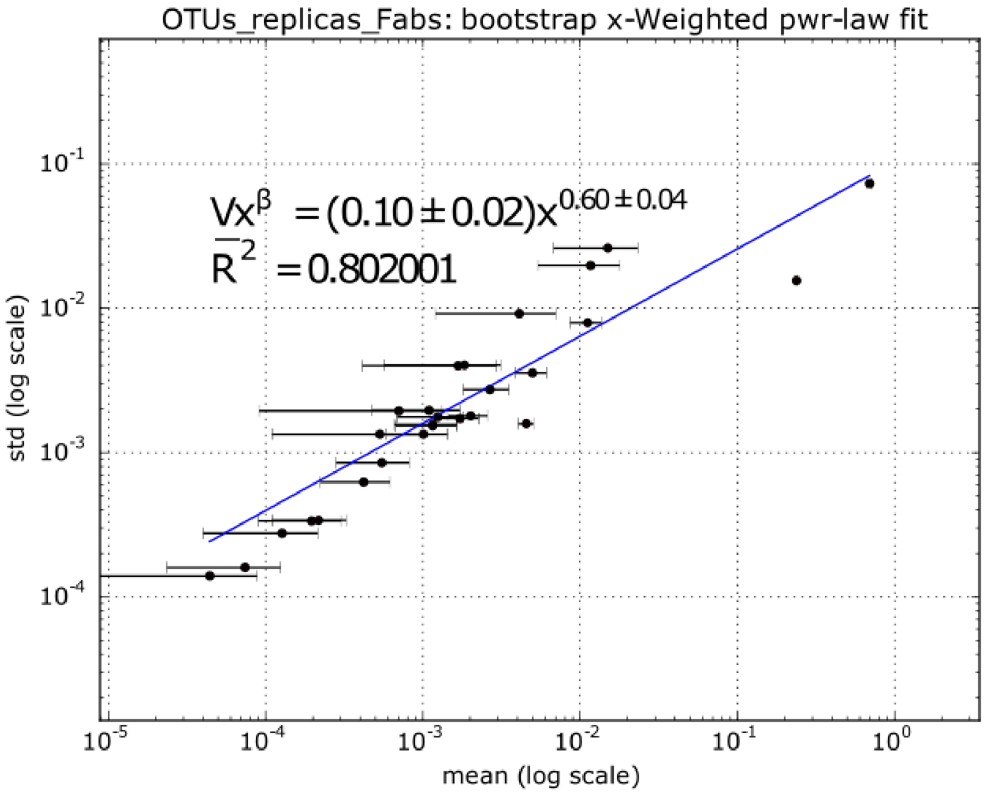

Supplementary Figure 2. Taylor law. We show the standard deviation as a function of the mean relative abundances for the 26 OTUs in the ten replica at the initial time sampled. Data follow a power law with a fluctuating scaling compatible with 0.5 (slope in log-log scale), as expected by Poisson-distributed replica. Variability of the Poisson sampling, including all sources up to taxonomic classification, is 8%, which can be interpreted as the expected variability as the number of taxa tends to one.

Supplementary Data. Plotting code used to generate the 3D representation of microbial consortia.

```
%%  scatterdistances3DMDS

function scatterdistances3DMDS(move)
    % I/O data paths
    inputPath = '/home/jocarbur/Ific/matlab/input';
    outputPath = '/home/jocarbur/Ific/matlab/output/';
    files = dir(fullfile(inputPath,'otu*'));

    if nargin < 1
        move = 0;
    end;

    % Default parameters
    visible='on';
    renderer='ZBuffer';
    speedCamera=2;
    viewAngle=-141;
    viewElevation=20;
    n=3;
    sizeRadius=100;

    % Calculate matrix random initial position
    R=rand(27,3);

    % For every file in directory
    for i = 1:length(files)
        baseFileName = files(i).name;
        fullFileName = fullfile(inputPath, baseFileName);
        s = load(fullFileName, 'fullMatrix');
        [nrow, col] = size(s);

        radiusDistances = s(1,:);
        matrixDistances = s(2:nrow,:);

        % Set max. number of iterations.
        opts = statset('Display','final','MaxIter',3000);

        [Y,stress] =
mdscale(matrixDistances,3,'Start',R,'criterion','sstres','Options',opt
s);

        dx = Y(:,1)';
        dy = Y(:,2)';
        dz = Y(:,3)';

        dx=dx+(-1.0*dx(1));
        dy=dy+(-1.0*dy(1));
        dz=dz+(-1.0*dz(1));

        % Put Colors

        negro=[0/255 0/255 0/255];
        morado=[.6 .0 1];
        azul_claro=[18/255 252/255 240/255];
        rojo=[205/255 6/255 17/255];
        naranja=[215/255 121/255 6/255];
```

```
        azul=[6/255 9/255 215/255];
        blanco=[.78 .80 0.96];
        amarillo=[255/255 255/255 0/255];

        grupo1=rojo
        grupo2=azul_claro
        grupo3=amarillo
        grupo4 =negro


        C = ones(1,1)*grupo4; % otu1 resto
        C = [C;ones(1,1)*grupo4]; % otu2 resto
        C = [C;ones(1,1)*grupo1]; % otu3 grupo1
        C = [C;ones(1,1)*grupo2]; %otu4 grupo2
        C = [C;ones(1,1)*grupo4]; %otu5 resto
        C = [C;ones(1,1)*grupo1]; %otu6 grupo1
        C = [C;ones(1,1)*grupo2]; %otu7 grupo2
        C = [C;ones(1,1)*grupo3]; %otu8 grupo3
        C = [C;ones(1,1)*grupo4]; %otu9 resto
        C = [C;ones(1,1)*grupo1]; %otu10 grupo1
        C = [C;ones(1,1)*grupo3]; %otu11 resto
        C = [C;ones(1,1)*grupo4]; %otu12 resto
        C = [C;ones(1,1)*grupo1]; %otu13 grupo1
        C = [C;ones(1,1)*grupo4]; %otu14 resto
        C = [C;ones(1,1)*grupo4]; %otu15 resto
        C = [C;ones(1,1)*grupo4]; %otu16 resto
        C = [C;ones(1,1)*grupo3]; %otu17 grupo3
        C = [C;ones(1,1)*grupo2]; %otu18 grupo2
        C = [C;ones(1,1)*grupo2]; %otu19 grupo2
        C = [C;ones(1,1)*grupo3]; %otu20 grupo3
        C = [C;ones(1,1)*grupo4]; %otu21 resto
        C = [C;ones(1,1)*grupo2]; %otu22 grupo2
        C = [C;ones(1,1)*grupo1]; %otu23 grupo1
        C = [C;ones(1,1)*grupo1]; %otu24 grupo1
        C = [C;ones(1,1)*grupo4]; %otu25 resto
        C = [C;ones(1,1)*grupo4]; %otu26 resto
        C = [C;ones(1,1)*grupo4]; %otu27 resto

        % Scatter Plot

hFigure=figure('name',baseFileName,'visible',visible,'Renderer',render
er, 'Units', 'pixels', 'Position', [0 0 600 600])
        hold on;

        radiusDistances=(radiusDistances/sizeRadius);
        scatter3sph(dx,dy,dz,'size', radiusDistances, 'color', C);

        colormap(jet);
        % Join Points
        size1=0.004
        size2=0.0105
        h0 = Cylinder([dx(6)-0.01 dy(6)+0.01 dz(6)-0.01],[dx(3) dy(3)
dz(3)],size2,50,[0.2 0.8 1],0,0);
        h1 = Cylinder([-1.093 1.159 -0.7543],[-1.188 1.125 -
0.72],size1,50,[0.2 0.8 1],0,0);
        %h0 = Cylinder([dx(6)-0.01 dy(6)+0.01 dz(6)-0.01],[dx(3) dy(3)
dz(3)],size2,50,[0.2 0.8 1],0,0);
        %h1 = Cylinder([-1.093 1.159 -0.7543],[-1.22 1.116 -
0.7113],size1,50,[1 0.27 0.27],0,0);
        % Put labels
```

```
labels={'1','2','3','4','5','6','7','8','9','10','11','12','13','14','
15','16','17','18','19','20','21','22','23','24','25','26','27'};
        dz_label=dz+radiusDistances+0.05;
        dx_label=dx+radiusDistances+0.05;
        dy_label=dy+radiusDistances+0.05;
        text(dx_label,dy_label,dz_label,labels, 'FontSize', 8,
'FontName','AvantGarde');

        set(gcf,'renderer','opengl');

        % Axis range
        axis equal; axis tight;
        view(viewAngle,viewElevation); grid ON;
        axis vis3d;
        material shiny;
        delete(findall(gcf,'Type','light'));
        camlight('left','infinite');
        lighting gouraud;

        % Write plot to file
        nameFileOutput=strcat(outputPath,baseFileName ,'.png');
        set(gca, 'Box','on','TickDir','out','TickLength',[.02
.02],'XMinorTick','on','YMinorTick','on','YGrid','on','XGrid','on','XC
olor',[.3 .3 .3],'YColor',[.3 .3 .3],'ZColor',[.3 .3
.3],'LineWidth',0.5);
        set(findobj('type', 'line'), 'color', [.3 .3 .3]);
        set(hFigure, 'PaperSize',[20 20]);
        set(hFigure, 'PaperPosition',[0 0 20 20]);
        set(gcf, 'PaperUnits', 'centimeters');
        set(gcf, 'PaperPositionMode', 'auto');
        %print(hFigure, '-dpng', '-painters','-r600', nameFileOutput);
        % Make movement
        if move>0;
            zoom(1.5);
            while ishandle(fig);
                camorbit(speedCamera,0.0);
                drawnow;
            end;
        end;
    end
```
